# Comparative genomics of *Eucalyptus* and *Corymbia* reveals low rates of genome structural rearrangement

**DOI:** 10.1186/s12864-017-3782-7

**Published:** 2017-05-22

**Authors:** J. B. Butler, R. E. Vaillancourt, B. M. Potts, D. J. Lee, G. J. King, A. Baten, M. Shepherd, J. S. Freeman

**Affiliations:** 1 School of Biological Science, University of Tasmania, Private Bag 55, Hobart, TAS 7001 Australia; 2 School of Biological Science and ARC Training Centre for Forest Value, University of Tasmania, Private Bag 55, Hobart, TAS 7001 Australia; 3 Forest Industries Research Centre, University of the Sunshine Coast, Locked Bag 4, Maroochydore DC, QLD 4558 Australia; 4 Southern Cross Plant Science, Southern Cross University, Military Rd, Lismore, NSW 2480 Australia

**Keywords:** Chromosome rearrangement, *Corymbia citriodora*, DArTseq, *Eucalyptus grandis*, Genome structure, Linkage mapping

## Abstract

**Background:**

Previous studies suggest genome structure is largely conserved between *Eucalyptus* species. However, it is unknown if this conservation extends to more divergent eucalypt taxa. We performed comparative genomics between the eucalypt genera *Eucalyptus* and *Corymbia*. Our results will facilitate transfer of genomic information between these important taxa and provide further insights into the rate of structural change in tree genomes.

**Results:**

We constructed three high density linkage maps for two *Corymbia* species (*Corymbia citriodora* subsp. *variegata* and *Corymbia torelliana*) which were used to compare genome structure between both species and *Eucalyptus grandis*. Genome structure was highly conserved between the *Corymbia* species. However, the comparison of *Corymbia* and *E. grandis* suggests large (from 1–13 MB) intra-chromosomal rearrangements have occurred on seven of the 11 chromosomes. Most rearrangements were supported through comparisons of the three independent *Corymbia* maps to the *E. grandis* genome sequence, and to other independently constructed *Eucalyptus* linkage maps.

**Conclusions:**

These are the first large scale chromosomal rearrangements discovered between eucalypts. Nonetheless, in the general context of plants, the genomic structure of the two genera was remarkably conserved; adding to a growing body of evidence that conservation of genome structure is common amongst woody angiosperms.

**Electronic supplementary material:**

The online version of this article (doi:10.1186/s12864-017-3782-7) contains supplementary material, which is available to authorized users.

## Background

Comparative genomics is a rapidly expanding field of research, with the potential to provide important evolutionary insights, as well as useful practical information [1–4]. For example, understanding the genomic similarities and differences between taxa is a central goal of evolutionary genetics, while the identification of conserved genome structure is important for inferring shared ancestry between taxa, and for the transfer of genetic information [5]. The increasing availability of genomic resources, such as genome sequences and high throughput molecular markers, now provides the opportunity for comparative genomics studies across an ever growing variety of taxa, yielding novel insights regarding the evolution of individual genes or gene families [6–8] through to entire genomes [9–11]. Species of economic importance such as grasses have been well studied in this regard, while trees have been relatively poorly studied.

Linkage maps are invaluable for the study of genome-wide structural variation between species, especially in the absence of an assembled genome [12]. Linkage maps are a genomic resource that have broad utility, including: the study of quantitative traits [13, 14]; comparative genomics [15]; analysis of recombination rate [16, 17]; and sequence assembly [18–20]. Genome structure comparisons can be performed by comparing several maps [15, 21], or comparing maps with assembled genomes [22–24].

Eucalypts are a group of trees belonging to the Myrtaceae family, containing the genera *Angophora, Corymbia* and *Eucalyptus* [25]. There are over 700 different species of eucalypts spanning 10 subgenera of *Eucalyptus* and two subgenera of *Corymbia* [26]. Most species belong to the *Eucalyptus* subgenus *Symphyomyrtus*, including many of economic importance such as *Eucalyptus grandis*, *E. urophylla* and *E. globulus* [27, 28]. *Eucalyptus grandis* is the reference genome for eucalypts [29]. Analysis of this genome provided evidence for a whole genome duplication (dated approximately 110 million years ago [MYA]) in eucalypts, which was suggested to have been pivotal in the evolution of the Myrtales and diversification from other Rosids [29]. The potential for further genomic studies in these important genera has been greatly enhanced by the release of this resource [30]. However, the efficacy of information transfer from this reference genome to other species will depend upon their similarity in genome structure, in terms of both synteny (the location of loci on homologous linkage groups) and collinearity (the congruent ordering of loci on homologous linkage groups). Early linkage mapping in eucalypts has allowed comparison of genome structure between *E. grandis* and other symphyomyrts such as *E. urophylla* [31], *E. globulus* [32], as well as *Corymbia* species [21], with each study reporting no strong evidence for structural differences. However, the relatively small number of markers used for map construction in these studies (such as SSRs and AFLPs) and the need for common markers between maps restricted the resolution of the comparisons that could be drawn. The development of high-throughput, sequence anchored markers in eucalypts has removed these limitations, allowing for much higher resolution genetic maps to be produced and the comparison of linkage maps directly to the reference genome [18, 33–37]. One recent study using such markers found support for two small inter-chromosomal translocations between *E. globulus* and *E. grandis* × *E. urophylla* hybrids [38]; one of which was supported by replication (independently constructed linkage maps) making it the most definitive genomic difference discovered in eucalypts. Aside from this, a high degree of genome conservation was assumed between members of *Symphyomyrtus* based on all past studies [28]. Only one study has performed comparisons outside of this subgenus into the more distant *Corymbia* [21], but was limited in the number of shared markers. With the advancement of marker technologies more comprehensive comparisons can be made between more divergent eucalypt taxa.

*Corymbia*, only recently classified as a separate genus to *Eucalyptus* [39], includes 113 species [40], with most endemic to the tropics, arid, and semi-arid zones of northern Australia [39]. Of these, *Corymbia citriodora* subsp. *variegata* (spotted gum) is a species with a prominent role in forestry both in Australia and overseas [41], where it is used for products including timber, charcoal and essential oil [41–43]. *Corymbia citriodora* subsp. *variegata* can readily hybridize with *C. torelliana*, an invasive tree species [44, 45] from the same subgenus but a different section of *Corymbia* [39, 40]. *Corymbia torelliana* is of interest to forestry due to the potential for increased growth rate in hybrids [46, 47]. *Corymbia citriodora* subsp. *variegata* and *C. torelliana* have estimated genome sizes of 370 MB and 390 MB, respectively [48], which is in contrast to the much larger *E. grandis* genome of 640 MB [48]. Despite these differences in genome size, both *Corymbia* and *Eucalyptus* share the same chromosome number, which is conserved across all eucalypts [48] and indeed across most Myrtaceous species [28]. *Corymbia* and *Eucalyptus* separated an estimated 52 MYA [49, 50], and the extent to which changes in genome structure have accumulated in that time and contributed to differences in genome size are unknown.

The extent of genomic differentiation between taxa, and the rate at which this accumulates, has important practical and evolutionary implications. These include influencing reproductive isolation as well as recombination in interspecific hybrids [51, 52]. There is increasing evidence that woody perennials are characterised by relatively slow rates of genomic change, whether at the level of substitution rate, chromosomal structure or ploidy [4, 53, 54]. For instance, a cytological study comparing various woody genera within the Fagaceae family found varying genome size, but no instances of polyploidy contributing to the diversification of this family [55]. Likewise, a comparative genomic study found a high amount of structural conservation between northern hemisphere trees from genera *Vitis*, *Populus*, *Malus* and *Juglans*, relative to herbaceous genera such as *Arabidopsis* and *Medicago* [53]. Indeed, comparisons between the genomes of herbs and grasses often reveal highly divergent structure, with studies detailing high chromosome fragmentation and ploidy changes [56–59]. However, high resolution comparative genomics studies have largely been restricted to a few tree families, such as Fagaceae [60], Pinaceae [61] and Salicaceae [62], therefore it is yet to be seen whether a reduced rate of genomic change compared to herbs is a characteristic of most trees.

In this study we compare the genome structure of the eucalypt genus *Corymbia* to that of *Eucalyptus*. Using 15,360 sequence-based Diversity Array Technology (DArTseq) markers and a marker binning technique [63, 64] we created high density linkage maps for *C. citriodora* subsp. *variegata* (CCV) and *C. torelliana* (CT). These maps were used to compare genome structure between each parental species and between these *Corymbia* species and *E. grandis* using the reference genome. We present evidence for differences in genome structure which are discussed in the context of the evolutionary relationships between the species and the stability of plant genomes through evolutionary time.

## Methods

### Genetic material

**Fig. 1:**
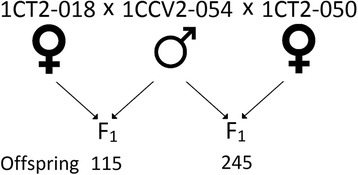
Design of the *Corymbia* pedigrees used to create the linkage maps. CT refers to *Corymbia torelliana*, while CCV refers to *Corymbia citriodora* subsp. *variegata*

Three genetic linkage maps were generated using two *Corymbia citriodora* subsp. *variegata* (CCV) × *Corymbia torelliana* (CT) F_1_ hybrid pedigrees (360 seedlings), resulting from a cross of the same CCV pollen parent (1CCV2-054) with two different CT parents (1CT2-018 and 1CT2-050, Fig. 1).

### DNA extraction protocol

Offspring were grown in glasshouse conditions until approximately 50 cm tall before sampling. Leaf samples were taken from each individual in the mapping family (including the parents) and dried over silica gel prior to DNA extraction. Total genomic DNA was extracted from 100 mg of dry leaf tissue using a QIAGEN (Hilden, Germany) DNeasy Plant Maxi Kit. The standard protocol was modified as follows: the volume of the AP1 buffer was increased to 1.5 × standard (i.e. 600 μl), 2% PVP-40 was added to the tissue lysis solution, and DNA was loaded onto the spin columns over two centrifugations before elution to increase yield. DNA samples were concentrated by vacuum drying and quantified using a PicoGreen assay (Molecular Probes, Eugene, OR). Samples were then adjusted to achieve a target concentration of 50 ng/μl by either dilution in 1× TE buffer, or further concentration using a sodium acetate precipitation, where appropriate. 15 μl of solution was supplied to DArT.

### DArTseq genotyping

Genotyping was performed by Diversity Array Technology Pty. Ltd. (Canberra, Australia) using DArTseq technology [65], which generates 64 base pairs (bp) of sequence at each marker by next generation sequencing. DArTseq yields two types of markers based on sequencing of genomic representations; co-dominant single nucleotide polymorphisms (SNP) and dominant markers which may represent SNP, or length polymorphisms in restriction enzyme recognition sites or restriction fragments. All test-cross (i.e. uniparentally segregating) markers were recoded into a double haploid configuration to allow a marker binning process to take place as SIMPLEMAP [63] requires population data in this format (see the Linkage map construction section). These markers were grouped into quality classes for the different mapping approaches using the following parameters (supplied by DArT PL.): reproducibility; call rate; and polymorphism information content (PIC). The latter is a measure of segregation ratios (a PIC of 0.5 indicates perfect 1:1 segregation) [66]. First class dominant markers featured reproducibility 1.0, call rate >95%, and PIC >0.35 (SNP markers featured average PIC >0.20); the second class featured reproducibility >0.9, call rate >90% and PIC >0.25 (SNP markers average PIC >0.15). A third class of markers, used only within bins, featured reproducibility >0.9, call rate >80% and PIC >0.15 (SNP markers average PIC >0.10, or markers with ambiguous or impossible segregation data, which was resolved by correcting offspring genotypes based on the segregation of the parents [assessed from three replicates of each parent]). Markers that did not meet these thresholds were excluded from further analysis. Fully informative inter-cross markers that segregated 1:1:1:1 were recoded into separate loci, each displaying the alleles segregating from a single parent (i.e. into the double haploid configuration required by SIMPLEMAP).

### Linkage map construction

The vast number of molecular markers provided by high-throughput technologies, such as DArTseq, challenges conventional approaches for linkage mapping. A marker binning process in SIMPLEMAP [63] was used prior to map construction to increase computational efficiency and improve the accuracy of high-density map construction [67]. In the binning process, a single representative marker is identified which represents a set of co-segregating and tightly linked markers in each bin (hereafter referred to as a ‘bin marker’). The bins are created according to a user defined maximum number of recombination events (the ‘repulsion threshold’) between any pair of markers. SIMPLEMAP recommends a maximum repulsion threshold equivalent to 3 cM for the Kosambi mapping function [63], which for small map distances is equivalent to a recombinant frequency of approximately 3% [68]. Therefore, a repulsion threshold of three and seven recombinants was used for cross 1CT2-018 × 1CCV2-054 and cross 1CT2-050 × 1CCV2-054 respectively, equivalent to a recombination frequency of less than less than 3% in each cross.

**Fig. 2:**
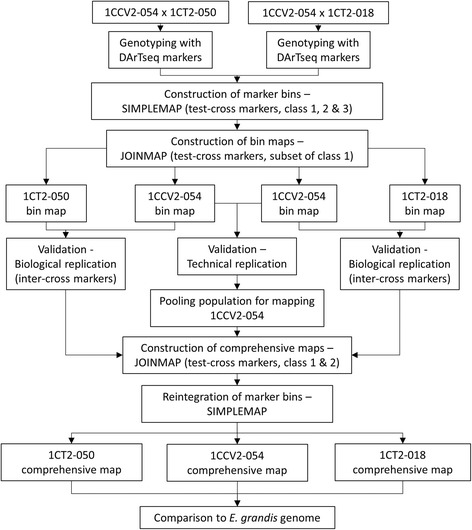
Summary of methods followed to create *Corymbia citriodora* subsp. *variegata* and *Corymbia torelliana* linkage maps

All mapping was undertaken using JoinMap v4 [69]. In summary, individual parental maps were initially constructed for both pedigrees using only bin markers (hereafter termed ‘bin maps’). These maps were used to assess biological and technical replication between the parental maps. Comprehensive parental maps were then constructed for each pedigree, using all markers to provide a higher resolution comparison against the *E. grandis* genome. A summary of the methods is presented below (Fig. 2).

### Parental bin map construction

Separate bin maps were created for both parents in each cross using only first class markers segregating 1:1. After removing markers and individuals with >10% missing data, markers were placed into linkage groups at a minimum of LOD 3. The regression algorithm and Kosambi mapping function [70] were used to order markers within linkage groups, using default JoinMap v4 settings. In an attempt to construct maps with robust marker order, an iterative approach was used and stringent criteria were imposed to evaluate map orders and remove problematic markers in each linkage group. Specifically, markers with a Chi-square goodness-of-fit contribution >1.0, or present in >1 double crossover were excluded. Markers with segregation distortion widely different from their closely-linked markers, were also excluded as these were likely to represent genotyping errors [69]. After removing markers according to these criteria, linkage maps were re-calculated and the above criteria were again evaluated. This procedure was repeated until threshold values were reached by all markers in each linkage group. Maps were then recreated using the Maximum Likelihood algorithm and compared with those created by the regression algorithm to verify marker order. To avoid interpreting potential error in the ordering of tightly linked markers as a departure from collinearity of syntenic markers (see the Discussion) section), a threshold of 1 cM was used to detect non-collinearity. Any shift in marker position exceeding this threshold between the maps was criteria for re-evaluation of marker statistics and further removal based on statistical support for marker order, until collinearity was established between maps produced using the different algorithms.

### Comparison of parental bin maps

In order to evaluate the repeatability of marker ordering, both biological and technical replication was evaluated by calculating Spearman’s correlations between linkage groups in different maps. Technical replication was evaluated by comparing the order of markers in each linkage group between the two independent maps of the male parent (1CCV2-054) which is shared between crosses. The technical replicates represent meiosis from the same genotype sampled in two different crosses. Biological replication was evaluated by comparing CCV and CT maps within crosses, as they represent different samples of meiosis and are from different genetic material (i.e. different species of *Corymbia*). The latter required mapping inter-cross (i.e. bi-parentally segregating, 1:2:1 and dominant 3:1) markers together with the test-cross (1:1) bin markers, to allow direct comparison of the parents within each pedigree based on common markers. When adding inter-cross markers, the ordering of markers in each round of mapping was evaluated as described above for bin maps, with removal biased towards retaining these bi-parentally segregating markers.

### Mapping of CCV using segregation data from the two different populations, and comprehensive map construction

Given the high correlation between CCV bin maps (see results below), genotype data from both populations was combined to map test-cross markers segregating from the CCV parent in both crosses, and this dataset was treated as a single population (*n* = 360). Due to the increase in sample size, markers were re-binned with SIMPLEMAP using a repulsion threshold of 10 recombinants to create bins spanning less than 3 cM.

Mapping of this combined CCV dataset (and the original CT datasets) was undertaken using only first and second class bin markers. After removing markers and individuals with >10% missing data, markers were added in an iterative fashion, starting with approximately 500 high quality markers, with batches of around 250 lower quality markers added in subsequent rounds. Markers were grouped at a minimum of LOD 3, and were ordered using the regression algorithm and Kosambi mapping function [70]. In each iteration, markers were removed according to the criteria described above, except the threshold for Chi-square goodness-of-fit contribution was raised to >2 after the initial 500 markers. Marker order was verified by comparing the final map from the previous round to the map produced using the Maximum Likelihood algorithm. Any shift in marker position exceeding 1 cM in any of these comparisons was criteria for re-evaluation of marker statistics and removal according to statistical support for marker order as above (in this case also considering the quality ranking of each marker), until collinearity was established.

Subsequently, ‘comprehensive maps’ were constructed, in which all markers (including first, second and third class) from the binning procedure were reintegrated into the bin maps. In SIMPLEMAP, reintegration of binned markers is performed using the percentage of recombinants between two markers to order the markers within bins around their representative bin marker, whose position is fixed [63].

### Comparison with *Eucalyptus grandis*

To compare the genome architecture of the *Corymbia* species and *E. grandis*, the marker sequences (length 64 bp) were searched against the *E. grandis* genome v2 [18, 29] to identify putative sequence homologs, using BLASTN [71]. For each marker the highest scoring hit (if multiple) was accepted only if it exceeded 95% of query coverage, and had an e-value < 1e^-10^. Markers that fell on unanchored *E. grandis* scaffolds were not considered. In order to examine synteny (the location of loci on homologous linkage groups) and collinearity (the congruent ordering of loci on homologous linkage groups) between *Corymbia* and *E. grandis*, the physical position of these hits in *E. grandis* were plotted against genetic position on the CCV map, and marker order was compared using Spearman’s rank correlation. Given the high collinearity of syntenic markers discovered between *E. grandis* and *Corymbia*, linkage group numbering and the orientation of linkage groups for the CCV and CT maps followed Brondani et al. [31], which corresponds to the chromosomes of the *E. grandis* reference genome [29].

To determine if there were any detectable instances of inter-chromosomal duplication involving multiple collinear markers in *E. grandis* relative to CCV, a second round of BLAST was undertaken allowing for multiple high scoring pairs per marker, and the position of these hits was compared to the CCV map as above.

## Results

### DArTseq genotyping

After preliminary data analysis to remove poor quality markers, DArTseq genotyping yielded 10,726 markers segregating 1:1 from the 1CCV2-054 individual, and 6,554 and 6,323 segregating 1:1 from 1CT2-050 and 1CT2-018, respectively, across the 3 quality classes described above (Additional file 1: Table S1). Dominant markers made up the bulk of the total, with co-dominant SNP markers averaging 25% of the markers across each individual.

### Comparison of parental bin maps

The bin maps for each parent in the two crosses comprised 340 to 446 bin markers. The rank order of the two bin maps of 1CCV2-054 (technical replicates) were highly correlated, providing strong support for the marker order (Table 1) and for the approach of combining the two populations to produce a comprehensive map of 1CCV2-054. Likewise, the high rank order correlation of the parental maps within pedigrees (biological replicates) provided good support for map order, and implied the genomes of the two *Corymbia* species are highly collinear. However, correlations could not be carried-out for linkage group 5 and 11 (Table 1) due to insufficient bi-parentally segregating markers.

**Table 1:** Spearman’s correlation of marker order in *Corymbia citriodora* subsp. *variegata* and *Corymbia torelliana* bin maps

Linkage Group	Spearman’s correlation^a^					
	1CCV2-054 vs 1CCV2-054		1CT2-050 vs 1CCV2-054		1CT2-018 vs 1CCV2-054	
	(bin markers)		(inter-cross markers)		(inter-cross markers)	
1	1.00***	(25)	0.80	(4)	0.93***	(9)
2	1.00***	(27)	1.00***	(3)	1.00***	(9)
3	0.99***	(19)	1.00**	(5)	0.96***	(14)
4	1.00***	(32)	1.00***	(4)	0.98***	(13)
5	1.00***	(31)	NA	(1)	1.00***	(7)
6	1.00***	(23)	0.99***	(17)	1.00***	(4)
7	0.99***	(21)	1.00**	(6)	1.00***	(4)
8	1.00***	(34)	1.00***	(7)	0.97***	(9)
9	1.00***	(28)	0.60	(5)	0.90*	(5)
10	1.00***	(23)	1.00***	(7)	1.00***	(11)
11	1.00***	(23)	0.94*	(6)	NA	(2)

^a^Numbers in brackets indicate the number of shared markers present between each bin map, while the type of marker is specified in the column heading. NA indicates linkage groups where less than three common inter-cross markers (dominant markers segregating 3:1 and SNP markers segregating 1:2:1) were able to be ordered, so no correlation was possible. *** *P* < 0.001, ** *P* < 0.01, * *P* < 0.05

### Comprehensive maps

The number of markers in the comprehensive maps ranged from 4,616–6,055, while map length ranged from 1,115–1,346 cM (Table 2, Additional file 2: Table S2). Marker density was high, with mean marker interval ranging from 0.26–0.61 cM. The map constructed for 1CT2-018 had the greatest length, mean and maximum marker interval, likely due to the relatively small population size used for map construction, as shown in a simulation by Bartholomé et al. [18]. The technical replicates created for 1CCV-054 support this observation, with the bin map created in the smaller pedigree also displaying a greater length, mean and maximum marker interval compared to the bin map from the larger pedigree (results not shown).

**Table 2:** Description of the comprehensive linkage maps generated for *Corymbia citriodora* subsp. *variegata* and *Corymbia torelliana*

Mapped individual		Population size	Length (cM)	Linkage group length (cM)	Markers	Unique positions	Mean interval between markers (cM)^a^	Maximum interval (cM)
1CCV2-054	♂	360	1179.9	77.2–137.6	6055	4510	0.26	10.5
1CT2-050	♀	245	1114.8	79.0–126.4	4689	2834	0.39	9.3
1CT2-018	♀	115	1345.6	94.8–158.7	4616	2212	0.61	15.5

^a^Mean marker interval was calculated from unique positions

### Collinearity of *Corymbia citriodora* subsp. *variegata* with *Eucalyptus grandis*

**Fig. 3:**
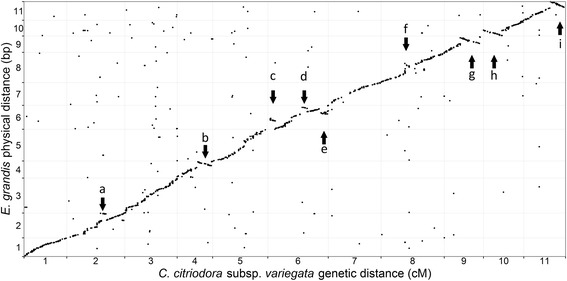
Marker positions in the *Corymbia citriodora* subsp. *variegata* comprehensive linkage map relative to the *Eucalyptus grandis* genome. Numbers along the x and y axis indicate the chromosome boundaries. Terminal inversions were detected in *C. citriodora* subsp. *variegata* relative to *E. grandis* on chromosomes 4, 9, 10 and 11; an intra-chromosomal translocation on chromosome 2; and more complex rearrangements on chromosome 6 and 8. The position of the above rearrangements are indicated by arrows, and named following Table 4. This figure was created using the package ‘ggplot2’ [103] in R [104]

Of the 6,055 markers ordered on the CCV comprehensive map, 1,441 were matched to a position on the *E. grandis* genome [29] at the threshold for acceptance (>95% query length, e-value < 1e^-10^_,_ highest scoring hit) (Fig. 3). Additionally, 204 CCV markers mapped to minor *E. grandis* scaffolds. Of the markers anchored to one of the 11 chromosomes, 165 (11%) were non-syntenic and 320 (22%) of the syntenic markers were non-collinear. Only markers that were at least 2 MB removed from the collinear order were declared as non-collinear to avoid interpreting possible error associated with ordering tightly linked markers as non-collinearity (see the Discussion section). There was a significant positive correlation in the order of syntenic markers between CCV and *E. grandis* (Table 3). Of the 1,441 markers which were placed on *E. grandis* chromosomes, 449 had more than one identically scored hit on the same chromosome, but as the majority of these were within 2 MB of each other and would have no impact on collinear order, one was selected at random. These multiple hits potentially reflect the numerous duplicate genes in tandem arrays known to be present in *E. grandis* [29].

**Table 3:** Marker order correlation between the *Corymbia citriodora* subsp. *variegata* map and the *Eucalyptus grandis* genome

Linkage Group	Spearman’s correlation^a^
1	0.99***
2	0.84***
3	0.95***
4	0.87***
5	0.98***
6	0.77***
7	0.98***
8	0.97***
9	0.71***
10	0.92***
11	0.79***

^a^Correlations below 0.95 are found on those chromosomes where putative rearrangements were found. *** *P* < 0.001

The analysis of collinearity provided evidence for nine major chromosomal rearrangements (involving consecutive non-collinear markers spanning >5 cM) between *E. grandis* and CCV, occurring on seven linkage groups. Specifically, large terminal inversions were evident on linkage groups 4, 9, 10 and 11, and more complex rearrangements detected on linkage groups 2, 6 and 8 (Table 4). To provide support for these putative rearrangements both of the maps generated for the CT parents were compared to the *E. grandis* reference genome (Additional file 3: Figure S1). Of the nine described rearrangements, seven were also present in both CT maps, while the rearrangements on linkage groups 2 and 8 could not be validated due to low marker density in these areas of the CT maps.

**Table 4:** Position of putative rearrangements in *Eucalyptus grandis* relative to *Corymbia citriodora* subsp. *variegata*

Chromosome^a^	Name^b^	Type	Position of markers flanking rearrangement (bp)^c^		Position of markers spanning rearrangement (bp)^d^	
2 (a)	CCV-in(2)tp1	Inversion/translocation	41313232	42473364	57510947	58000349
4 (b)	CCV-in(4)1	Inversion	25483501	40126737	29983251	38988874
6 (c)	CCV-in(6)tp1	Inversion/translocation	Start	2162368	20167425	24639366
6 (d)	CCV-in(6)tp2	Inversion/translocation	35523433	42332855	50159071	52725864
6 (e)	CCV-in(6)tp3	Inversion/translocation	48934608	56404168	36780165	39438890
8 (f)	CCV-in(8)1	Inversion	22156661	45300454	33441444	42114915
9 (g)	CCV-in(9)1	Inversion	20700201	37191595	21585756	33994985
10 (h)	CCV-in(10)1	Inversion	Start	13232993	1214529	14051146
11 (i)	CCV-in(11)1	Inversion	29255951	End	31407473	44623976

^a^The letter assignment corresponds to the naming of the rearrangement in Fig. 3

^b^Designation of each inversion. ‘in’ refers to an inversion, the number in brackets refers to the linkage group the rearrangement is localized to, ‘tp’ indicates the rearrangement is transposed within the chromosome, and the final number indicates occurrence on the chromosome, if multiple

^c^Refers to first marker on either side of the rearrangement. No flanking marker position was available if a rearrangement spanned the first or last marker on a linkage group in the *C. citriodora* subsp. *variegata* comprehensive map

^d^Refers to the marker in the first and last position of the rearrangement. Note, for translocations the position of markers flanking a rearrangement indicates the origin of the translocated region in *E. grandis*, while the position of markers spanning the rearrangement indicates the current configuration in the *C. citriodora* subsp. *variegata* comprehensive map

To investigate the possibility that the putative rearrangements were artefacts caused by errors in the *E. grandis* genome assembly, the areas of the *E. grandis* genome containing the nine putative rearrangements were checked to ensure collinearity with independently constructed high density linkage maps constructed in *E. grandis* and *E. urophylla* [18]. For this purpose, the physical location in *E. grandis* of all putative rearrangements (including two markers flanking the rearrangement) was assessed for collinearity with the genetic linkage maps (J. Bartholomé pers. comm.). These areas of the *E. grandis* genome were highly correlated with the marker order in both the *E. grandis* and *E. urophylla* linkage maps (Additional file 4: Table S3), giving confidence that these areas of the genome were assembled correctly.

The nine major intra-chromosomal rearrangements described above involved 200 (14%) of the 1,441 CCV markers placed on the *E. grandis* chromosomes. The remaining 120 (8%) non-collinear markers detected in this comparison were mostly singletons, but also included small clusters of tightly linked markers none of which spanned more than 5 cM (Fig. 3; Additional file 2: Table S2). Similarly, while distributed genome-wide, the majority of the 165 non-syntenic markers also occurred as singletons or in near identical positions to other markers, with no consecutive markers spanning more than 5 cM (Fig. 3; Additional file 2: Table S2), suggesting no major inter-chromosomal rearrangements have occurred between these species. Likewise, no instances of inter-chromosomal duplications involving multiple collinear markers were detected when examining markers with multiple matches on the *E. grandis* genome (Additional file 5: Figure S2).

## Discussion

We perform the first detailed comparisons of genome structure between *Corymbia* species, as well as between each species and the *Eucalyptus grandis* reference genome. The results of these comparisons provide the first evidence for large scale chromosome rearrangements in eucalypts. Previous comparative studies of eucalypts have pointed to largely conserved genome structure [28, 72]. However, detailed comparisons have been restricted to a few species within subgenus *Symphyomyrtus* [34, 35, 37, 38]. Comparison of *C. torelliana* and *C. citriodora* subsp. *variegata* linkage maps (both directly and *via* comparison of these linkage maps with the *E. grandis* genome), suggests genome structure is largely conserved between these *Corymbia* species. These species represent separate sections within *Corymbia* [39], so in terms of taxonomic distance are comparable to the previous inter-sectional comparisons within *Symphyomyrtus* [32, 37, 38]. In contrast, much greater genomic differentiation was evident in our comparison between the closely related genera *Eucalyptus* and *Corymbia*. Together with past findings our results provide further evidence that genome structure is highly conserved between closely related eucalypt species with more pronounced genomic differentiation found with increasing taxonomic distance.

Despite rearrangements being detected on seven linkage groups, the genomic structural differentiation found between the two genera in this study is low in the context of many plant taxa, such as *Arabidopsis, Sorghum*, *Zea*, *Brassica* and *Fragaria* [56–59], but comparable with the high level of genomic stability reported in other woody angiosperms. For example, while *Salix* (willow) and *Populus* (poplar) diverged approximately 45 - 52 MYA, comparative mapping [73, 74], and comparison of assembled genomes [75] reveal high synteny and collinearity between the two genera. Likewise, *Castanea* (chestnut) and *Quercus* (oak) diverged approximately 70 MYA, but comparative mapping based on 397 shared markers revealed conserved chromosome number and high collinearity [60]. In contrast, grasses and herbaceous plants often display chromosome reshuffling and changes in ploidy level between more recently diverged species [76]. Ploidy is stable throughout the Myrtaceae [28] and most other trees, with some exceptions [77, 78]. Although woody angiosperms do not form a single evolutionary lineage, shared characteristics such as their large size and longevity influence their mode and tempo of evolution [79] and this may extend to genome structure [60, 62]. Specifically, our findings from a geographically and phylogenetically independent angiosperm lineage from those in previous comparisons support the hypothesis that conservation of genome structure is a key evolutionary characteristic of trees [53, 55].

There are several potential explanations for conservation of genomic structure amongst diverse woody angiosperms. The disparity in the rate of genome structural changes between herbaceous and non-herbaceous plants may simply reflect differences in generational time, with more rapid genomic differentiation occurring in organisms with faster generation turnover relative to woody perennials [53, 80]. Further, Chen et al. [55] proposed that participation in syngameons (populations of different species with interbreeding) may play an important role in the conservation of genome structure in woody angiosperms. The premise is that syngameous relationships may promote genome conservation because inter-specific gene flow can be advantageous, potentially allowing rapid adaptation without the need for major genomic changes. Indeed, hybridisation has long been hypothesised to play an important role in eucalypt evolution [81–84]. Hybridisation in eucalypts is more frequent between closely related species and drops off sharply with increasing taxonomic distance [85–87]. For example, the symphyomyrts *E. grandis*, *E. urophylla* and *E. globulus* can all interbreed, as can the two *Corymbia* species in this study, consistent with the apparent conservation of genome structure between species within each of these genera [32, 38]. However, *Eucalyptus* and *Corymbia* do not hybridise with one another [82]. Assuming that interspecific hybridisation does contribute to genome conservation in closely related eucalypt species, one can speculate that the bulk of the putative rearrangements between *Eucalyptus* and *Corymbia* would have been selected against in a syngameous relationship, and may have occurred after these lineages were reproductively isolated. However, further study is required to better understand the evolution of genome structure between these genera and in eucalypts more broadly, ideally performing comparative genomics and phylogenetic analysis of several taxa representing different lineages.

The expansion and contraction of gene families by tandem duplication is another potential factor which may contribute to taxonomic differentiation amongst eucalypts while conserving gross genome structure [88–90]. Tandem duplication is thought to be a major mechanism creating new genes with implications for adaptation and speciation [91–93]. This may be particularly true in eucalypts, as *Eucalyptus grandis* has the largest proportion of genes in tandem repeats among sequenced plant genomes. Indeed, preliminary analysis points to variation in copy number of tandem repeats in comparison with the closely related *Eucalyptus globulus* [29], providing support for the role of tandem duplication in eucalypt diversification.

The use of a marker binning technique, iterative rounds of mapping and stringent thresholds for accepting a given map order contributed to very robust marker orders, as evidenced by the strong correlations between maps in this study. To our knowledge, these are the highest density linkage maps published in eucalypt to date [18, 36]. Establishing the correct map position of tightly linked markers in high-density linkage maps is statistically challenging [67, 94]. To alleviate this problem a marker binning technique was employed, which grouped tightly linked markers into bins before ordering. This was effective in reducing the computational complexity of mapping thousands of markers, and should have reduced gross errors which occur more frequently when attempting to order tightly linked markers [67]. As genotyping errors and missing data are also key factors producing incorrect marker order, particularly as marker density increases [94], our iterative approach of progressively increasing marker density from the highest quality markers (which generally contain the least genotyping errors and missing data) to those of lower quality gave us confidence in the marker orders produced and permitted an assessment of repeatability of marker orders.

The creation of individual parental maps based predominantly on test-cross markers also contributes to robust orders. Past studies have often employed bi-parental consensus maps incorporating inter-cross markers [33, 34, 38]. Such consensus maps have the advantage of allowing comparison of male and female maps and the location of QTL, and can result in increased marker density. However, a consensus map may be less robust, as inter-cross markers have been shown to reduce the accuracy of marker ordering [18]. Indeed, in this study when attempting to add inter-cross markers to compare the parental maps, only a few could be ordered at the required stringency. Further, merging parental maps can create errors due to heterogeneity between individuals used for map construction [95]. One of the main outcomes we sought to achieve through the creation of these maps was to inform a *Corymbia* genome assembly [96], along with facilitating further comparative genomics among eucalypts. Therefore, we chose to use individual parental maps with an emphasis on stringent marker order, rather than maximising the number of markers placed on a single map.

Both linkage maps and genome assemblies are prone to errors [18, 67, 69, 97] and should be independently validated where possible, in order to draw robust conclusions in comparative studies. However, studies of this nature rarely have replication. In our case, the majority of the rearrangements (seven out of nine) we describe are supported by independently constructed linkage maps in this study, providing replication; both within *C. citriodora* subsp. *variegata* and in a separate species, *C. torelliana*. The areas of the *E. grandis* genome assembly in which these putative rearrangements lie have also been validated through comparison of the *E. grandis* genome to independently constructed linkage maps [18]. As such, we are confident these putative rearrangements reflect real genomic differences between the taxa in question, rather than errors in linkage map construction or genome assembly.

Aside from the nine relatively large rearrangements, many smaller regions were non-syntenic or non-collinear in the comparison of the CCV linkage map and the *E. grandis* genome. These regions were dispersed throughout each genome with the majority represented by single markers, but also included small groups of (up to 5) markers. The placement of these markers likely represents both small genomic differences and analytical causes. In the case of the latter, despite the use of replication and stringent methodology errors may occur due to factors such as incorrect order [67] or linkage group assignment of mapped markers; errors in the *E. grandis* genome assembly [18]; and failure of BLAST to locate the true *E. grandis* homolog of markers in the CCV map. On the other hand, some differences are likely to reflect biological causes including small scale inversions, duplications and deletions as well as transposable element activity between the genomes, which have been implicated in inter-chromosomal rearrangements in eucalypts [38] and other taxa [98, 99]. In eucalypts an increasing level of small scale non-synteny was noted when comparing taxa with increasing taxonomic separation [38], so the level of non-synteny shown between these genera is not unexpected. Despite the fact that some of the apparent small genomic differences no doubt represent errors, overall, the linkage maps created in this study provide valuable insights into the extent of genome differentiation between *E. grandis* and *Corymbia* and highlight potential differences for further research.

Researchers are currently using the *E. grandis* reference genome for gene discovery across many eucalypt species while assuming conservation of genome structure, but our findings show this requires validation, particularly in divergent lineages such as *Corymbi*a. Large stretches of conserved marker orders were found between the genomes of *Corymbia* and *E. grandis*, with even those areas encompassed by putative rearrangements maintaining a conserved order within the inversions. These findings suggest that information regarding broad scale genomic features will be readily transferable between the two genera. However, transfer of information at the genic scale, such as the content and order of annotated genes [29, 100, 101], as well as the potential impact of expansion and contraction of genes in tandem arrays in *Corymbia*, will require further analyses at the sequence level. The putative rearrangements revealed in this study are likely to be of relevance to these analyses.

## Conclusions

In conclusion, this study provides a significant contribution to eucalypt comparative genomics, by examining differentiation between *Corymbia* species and *E. grandis*. The results reported here are the first glimpses into the changes that have occurred between two eucalypt genera since their divergence. Our experimental design and stringent methodology provides compelling evidence for chromosomal rearrangements between these genera. Despite these rearrangements our findings, together with past studies, suggest woody plants are characterised by a low rate of structural evolution in comparison to grasses and other herbaceous genera. The linkage maps constructed in this study have been crucial in the *de novo* assembly of the CCV genome [96], which has allowed more detailed comparative analysis of individual gene families [102], both of which will be reported in subsequent studies.

## Additional files

Additional file 1: Table S1. Summary of the number of test-cross markers available for mapping in each parent of the *Corymbia* pedigrees by class and type. (XLSX 9 kb)

Additional file 2: Table S2. Comprehensive linkage maps created for each parent of the *Corymbia* pedigrees. a) *Corymbia citriodora* subsp. *variegata* (1CCV2-054) b) *Corymbia torelliana* (1CT2-050) c) *Corymbia torelliana* (1CT2-018). (XLSX 1482 kb)

Additional file 3: Figure S1. Marker position in the *Corymbia torelliana* maps ((a) 1CT2-050 and (b) 1CT2-018) relative to the *Eucalyptus grandis* genome. (PNG 459 kb)

Additional file 4: Table S3. Correlation of putatively rearranged areas of the *Eucalyptus grandis* genome with independently constructed linkage maps in *E. grandis* and *E. urophylla*. (XLSX 9 kb)

Additional file 5: Figure S2. Duplicate marker position in the *Corymbia citriodora* subsp. *variegata* map relative to the *Eucalyptus grandis* genome. An e-value threshold of 1e^-10^ gave approximately 6000 high scoring pairs, which were allowed to be matched to multiple positions. Visual inspection of dot matrixes revealed no series of collinear markers that were represented on multiple chromosomes, suggesting no instances of inter-chromosomal duplications in *E. grandis* relative to *Corymbia*. (PNG 217 kb)
