# Supplementary figures and images for: Comparative genomics of Eucalyptus and Corymbia reveals low rates of genome structural rearrangement
Source: BMC Genomics. 2017 May 22;18:397. doi: 10.1186/s12864-017-3782-7 (PMC5441008; doi:10.1186/s12864-017-3782-7)

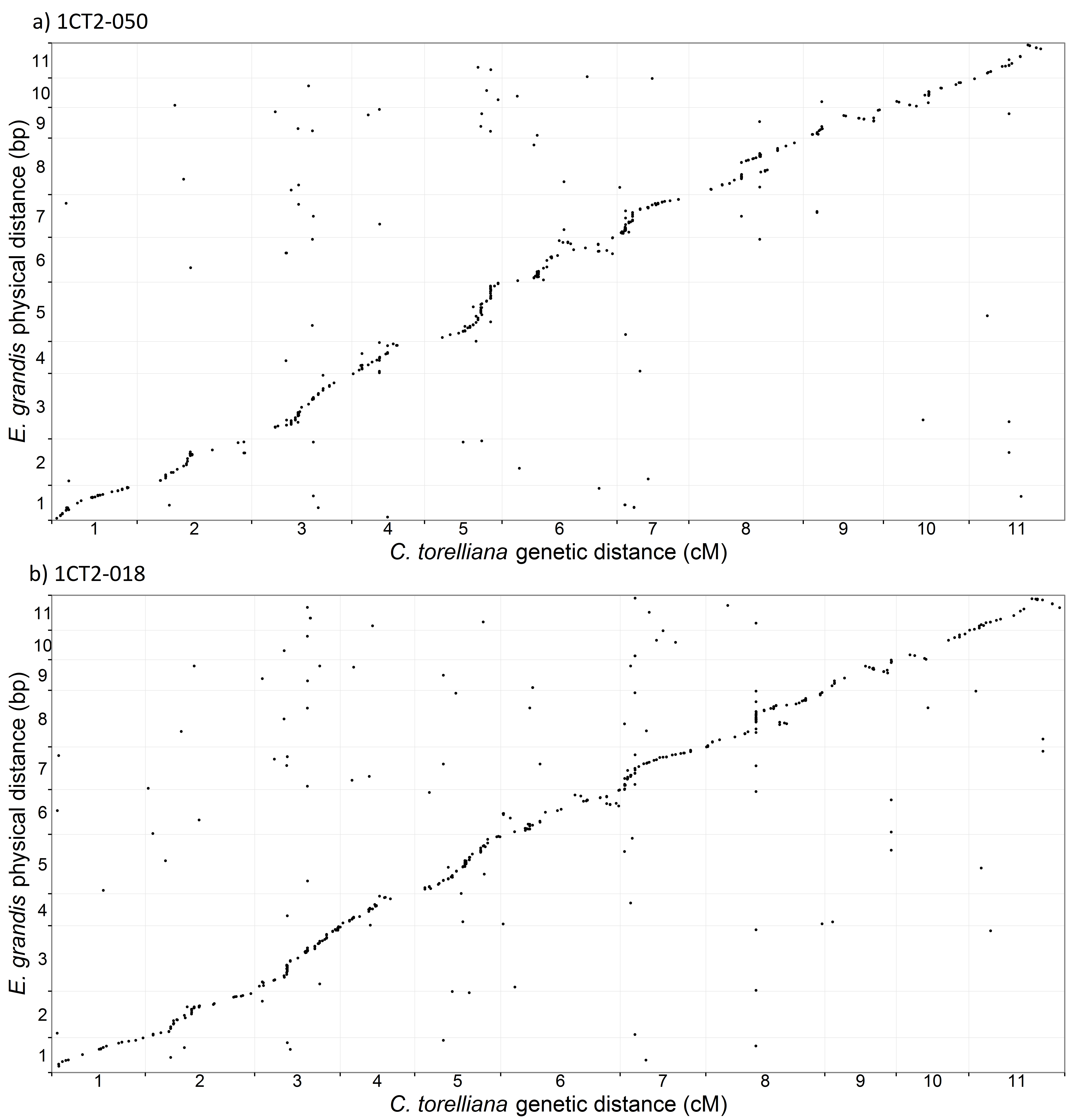

Supplement: Supplementary file 3 — Marker position in the Corymbia torelliana maps ((a) 1CT2-050 and (b) 1CT2-018) relative to the Eucalyptus grandis genome. (PNG 459 kb) [file 12864_2017_3782_MOESM3_ESM.png]

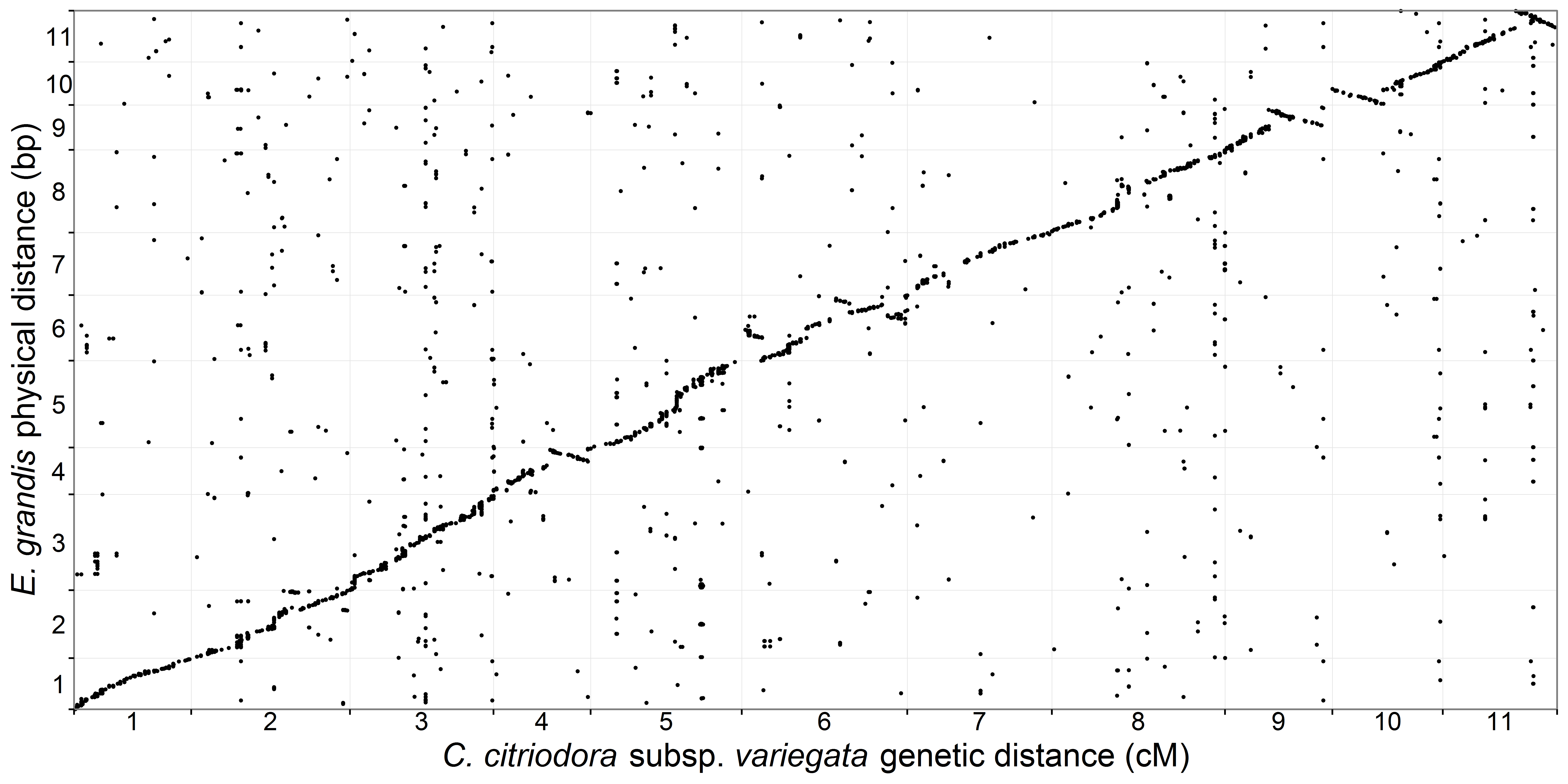

Supplement: Supplementary file 5 — Duplicate marker position in the Corymbia citriodora subsp. variegata map relative to the Eucalyptus grandis genome. An e-value threshold of 1e-10 gave approximately 6000 high scoring pairs, which were allowed to be matched to multiple positions. Visual inspection of dot matrixes revealed no series of collinear markers that were represented on multiple chromosomes, suggesting no instances of inter-chromosomal duplications in E. grandis relative to Corymbia. (PNG 217 kb) [file 12864_2017_3782_MOESM5_ESM.png]
